# Electroencephalographic characteristics of children and adolescents with chronic musculoskeletal pain

**DOI:** 10.1097/PR9.0000000000001054

**Published:** 2022-12-22

**Authors:** Don Daniel Ocay, Elizabeth F. Teel, Owen D. Luo, Chloé Savignac, Yacine Mahdid, Stefanie Blain-Moraes, Catherine E. Ferland

**Affiliations:** a Department of Experimental Surgery, McGill University, Montreal, QC, Canada; b Department of Clinical Research, Shriners Hospitals for Children-Canada, Montreal, QC, Canada,; c Department of Health, Kinesiology, & Applied Physiology, Concordia University, Montreal, QC, Canada; d Faculty of Medicine, McGill University, Montreal, QC, Canada; e Integrated Program in Neuroscience, McGill University, Montreal, QC, Canada; f Montreal General Hospital, McGill University Health Centre, Montreal, QC, Canada; g School of Physical and Occupational Therapy, McGill University, Montreal, QC, Canada; h Department of Anesthesia, McGill University, Montreal, QC, Canada; i Research Institute-McGill University Health Centre, Montreal, QC, Canada; j Alan Edwards Research Center for Pain, McGill University, Montreal, QC, Canada

**Keywords:** Pediatric pain, Clinical pain assessment, Chronic musculoskeletal pain, Electroencephalography, Sensory testing, Noninvasive neuroimaging

## Abstract

**Introduction:**

The pathophysiology of pediatric musculoskeletal (MSK) pain is unclear, contributing to persistent challenges to its management.

**Objectives:**

This study hypothesizes that children and adolescents with chronic MSK pain (CPs) will show differences in electroencephalography (EEG) features at rest and during thermal pain modalities when compared with age-matched controls.

**Methods:**

One hundred forty-two CP patients and 45 age-matched healthy controls (HCs) underwent a standardized thermal tonic heat and cold stimulations, while a 21-electrode headset collected EEG data. Cohorts were compared with respect to their EEG features of spectral power, peak frequency, permutation entropy, weight phase-lag index, directed phase-lag index, and node degree at 4 frequency bands, namely, delta (1–4 Hz), theta (4–8 Hz), alpha (8–13 Hz), and beta (13–30 Hz), at rest and during the thermal conditions.

**Results:**

At rest, CPs showed increased global delta (*P* = 0.0493) and beta (*P* = 0.0002) power in comparison with HCs. These findings provide further impetus for the investigation and prevention of long-lasting developmental sequalae of early life chronic pain processes. Although no cohort differences in pain intensity scores were found during the thermal pain modalities, CPs and HCs showed significant difference in changes in EEG spectral power, peak frequency, permutation entropy, and network functional connectivity at specific frequency bands (*P* < 0.05) during the tonic heat and cold stimulations.

**Conclusion:**

This suggests that EEG can characterize subtle differences in heat and cold pain sensitivity in CPs. The complementation of EEG and evoked pain in the clinical assessment of pediatric chronic MSK pain can better detect underlying pain mechanisms and changes in pain sensitivity.

## Abstract 2

Supplemental Digital Content is Available in the Text.

## 1. Introduction

The pathophysiology of pediatric musculoskeletal (MSK) pain is not fully understood, contributing to challenges in its management,^23^ eliciting significant burdens and leading to continued pain and disability in adulthood.^47^

Advances in noninvasive brain monitoring devices, such as electroencephalography (EEG), present opportunities to better characterize the neurological processes underlying pediatric chronic pain. An EEG biomarker of pain may pose as an advantage to the assessment of pain in youth with intellectual and developmental disabilities or as a target for noninvasive brain stimulation.^19,44^ Previous studies have identified adults with and without chronic pain showing distinct EEG patterns.^15,25,67^ Adult patients demonstrate increased resting EEG theta power (4–8 Hz) and decreased peak frequency to lower frequencies.^61^ Studies have also identified that pain connectome changes among adult patients,^42,74^ through altered functional connectivity between the EEG captured over different scalp areas, and that anesthetic gases alter permutation entropy, a measure of EEG information content quantifying the regularity of the continuous EEG time series.^36,56^

However, the pediatric neuroimaging literature has concentrated on employing fMRI in adolescents with complex regional pain syndromes (CRPS),^24,41,66,71^ identifying that pediatric and adult CRPS patients show different patterns of functional connectivity changes.^80^ This finding supports studies identifying age-dependent developmental changes in pediatric pain processing, perception, and responses.^2,7,17,30^ There is substantial impetus for extending the findings of the adult chronic pain EEG literature to pediatric populations. This study used EEG to interrogate brain activity and connectivity changes, previously observed in adult studies,^66,74^ in youth with chronic MSK pain at rest and during thermal experimental pain modalities (tonic heat and cold pressor task). We hypothesized that youth with chronic MSK pain will show developmental patterns of EEG neural activity and connectivity differences to age-matched pain-free controls at rest and an increased sensitivity to acute tonic pain experiences during thermal experimental pain modalities.

## 2. Materials and methods

### 2.1. Participants

Participant recruitment occurred between October 2018 and June 2021 from the orthopedic outpatient clinics and from the Chronic Pain Services of our institution. Inclusion criteria for patients include being between 10 and 18 years old, reporting MSK pain at least once weekly and lasting 3 months or longer, and able to understand and respond to the outcome measures. Children unable to speak, write, or read English or French, with pain due to an acute trauma occurring in the past 3 months (eg, fracture) or diagnosed with developmental delay or any severe systemic disease with some functional limitations, were excluded. Age-matched healthy controls (HC) with no chronic pain in the past 3 months were screened^27^ and recruited through word of mouth and recruitment advertisements. Ethics approval was obtained before the beginning of the recruitment from the Research Ethics Board of McGill University (A09-M17-17B). Participants provided written informed consent before inclusion in the study, and a signature was obtained from the participant or their parent/legal guardian, if they were younger than 14 years old, before beginning the study.

### 2.2. Sociodemographic characteristics and pain history

Demographics of all participants were collected through face-to-face interviews by a research assistant. All CP participants described their medical history and the location(s), duration, and frequency of their pain. The Douleur Neuropathique 4 (DN4) questionnaire was used to assess the neuropathic component of their pain.^10^ Before the assessment, all participants verbally rated their current pain intensity with a numerical rating scale (NRS) ranging from 0 (no pain) to 10 (worst pain imaginable).

The Pain Catastrophizing Scale for Children (PCS-C), Pittsburgh Sleep Quality Index (PSQI), and Revised Child Anxiety and Depression Scale (RCADS) were completed to assess the degree to which participants experienced negative thoughts or feelings while experiencing pain,^18,60^ sleep quality,^14,50,63^ and participants' self-report of depression and anxiety,^16^ respectively.

### 2.3. Thermal experimental pain modalities

Each participant underwent a tonic heat stimulus with a 9 cm^2^ warm calibrated thermode connected to a Q-sense apparatus (Medoc, Israel) placed on the right volar forearm and set to a predetermined test temperature eliciting a 50/100 pain intensity rating for the individual participant, using a computerized visual analogue scale (CoVAS) scale ranging from 0 (no pain) to 100 (worst pain imaginable). The temperature remained constant for 120 seconds and was blinded to the participants. To avoid expectation effects, participants were told that the temperature could increase, remain stable, or decrease and to evaluate their pain with the CoVAS throughout the test. The average pain intensity during the tonic heat stimulus was calculated. Each participant then performed a cold pressor task (CPT), with complete immersion of their left forearm in cold water (12°C) for 2 minutes while rating their pain with a NRS 0 to 10 every 15 seconds. If a participant removed their arm before the end of the 120 seconds, an average pain intensity score of 10/10 was given.

### 2.4. Electroencephalography recording

Brain activity was recorded with a dry EEG headset (DSI-24, Wearable Sensing) using 21 electrodes located at standard 10-20 system coordinates, sampled at 300 Hz, and referenced to Pz. Recordings were performed at resting state with eyes opened and during the thermal experimental pain modalities (tonic heat and cold pressor task conditions). Two different baseline recordings were conducted on 2 groups of participants: resting state with eyes open without or while moving the CoVAS to control for the motor aspect of the thermal heat pain modality.

### 2.5. Electroencephalography preprocessing

The EEG was preprocessed in EEGlab,^22^ band-pass filtered between 0.1 and 50 Hz, and rereferenced to A1 and A2 electrodes resulting in 19 EEG channels corresponding to the Fp1/2, F3/4/7/8/z, C3/4/z, T3/4/5/6, P3/4/z, and O1/2 electrodes. Independent component analysis^22,37^ was performed to remove artifacts of eye movement and facial muscle activity. The data were visually inspected, and the remaining bad segments were manually removed. The cleaned EEG data were segmented into 3 conditions of interest: resting state, tonic heat, and the CPT. Then, EEG segments were exported in a custom MATLAB plug-in EEGapp (EEGapp, BIAPT lab, McGill University) for analysis.^46^

### 2.6. Electroencephalography feature extraction

**Figure 1.**
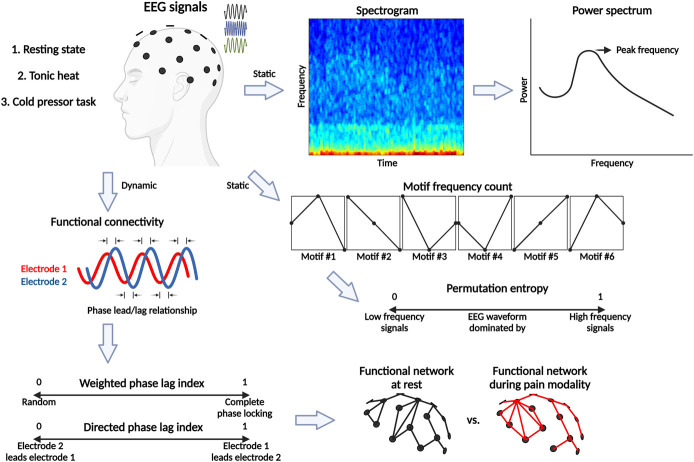
Analyses for EEG feature extraction. Participants underwent EEG recording at rest with eyes opened, and during thermal experimental pain modalities (tonic heat and cold pressor task conditions). Following preprocessing (not pictured here), EEG signals were used to compute the following measures within 4 frequency bands (delta, theta, alpha, and beta) on 10-second EEG segments. Global spectral power was calculated for each participant by averaging the spectral power across all channels within each frequency band. Global peak frequency was identified as the frequency with the largest power amplitude within each frequency range of interest. Permutation entropy was calculated by fragmenting the continuous EEG waveform into a sequence of motifs according to their shape (slopes, peaks, and troughs) and generating a probability distribution of their representation in the EEG. A normalized permutation entropy value approaching 1 indicates that the EEG waveform is dominated by higher-frequency signals, whereas a normalized permutation entropy value approaching its theoretical minimum of 0 suggests that the EEG waveform is primarily composed of low-frequency signals. Functional connectivity was measured by weighted phase lag index (wPLI) and directed phase lag index (dPLI). A wPLI value close to 1 indicates complete phase locking between the 2 EEG signals. Conversely, a wPLI value of 0 indicates that the phase lead/lag relationship between the signals is random. A dPLI value between 0.5 and 1 indicates that the EEG signal from electrode 1 leads the signal from electrode 2. Conversely, a dPLI value between 0 and 0.5 indicates that the EEG signal from electrode 2 leads the signal from electrode 1, and a dPLI value of 0.5 indicates that there is no phase lead or lag relationship between the signals. Graph analysis was used to characterize the functional connectivity network during the thermal experimental pain modalities in comparison at rest by measuring the total number of other electrodes to which a given electrode was functionally connected. Figure created in BioRender.com. EEG, electroencephalography.

All EEG features were calculated at 4 frequency bands—delta (1–4 Hz), theta (4–8 Hz), alpha (8–13 Hz) and beta (13–30 Hz)—on 10-second EEG segments. The gamma frequency band (30–200 Hz) was excluded because the skull acts as a natural filter, and gamma oscillations are usually recorded using high-density EEG.^1^ Six EEG features were calculated: spectral power, peak frequency, permutation entropy, weighted phase-lag index, directed phase-lag index, and node degree (Fig. 1).

To measure oscillatory neural activity,^31^ the spectral powers of each channel were calculated on the average spectrogram for a given window using the *spectopo* function in EEGlab.^22^ Spectrograms across all channels were calculated using the multitaper method with 3 tapers and a time bandwidth product of 2; global spectral power was calculated by averaging the spectral power across all channels within each frequency band. Global peak frequency was identified as the frequency with the largest power amplitude within each frequency range.

Permutation entropy was calculated by fragmenting the continuous EEG waveform into motifs (slopes, peaks, and troughs) and generating a probability distribution of their representation in the EEG with 2 parameters, embedding dimension (*d*_*E*_*)* and time delay (τ).^4^ We used *d*_*E*_ *=* 5 and τ = 4 to provide a sufficient deployment of the trajectories within the state space of the EEG beta activity.^35^ Global permutation entropy was calculated by averaging the permutation entropy across all channels within each frequency band.

To characterize the neural communication processes detected as the relationships between EEG signals measured by electrodes overlying neighboring cortical areas, functional connectivity was estimated with the weighted phase lag index (wPLI).^76^ To characterize the temporal precedence between 2 EEG signals, directed functional connectivity was estimated with the directed phase lag index (dPLI).^72^ Both the wPLI and dPLI between every pair of electrodes was computed, resulting in a 19 × 19 channel connectivity matrix, with each single value corresponding to the strength of connection between the cortical activity detected by 2 channels over the 10-second EEG segments. The average wPLI and dPLI functional connectivity measures was calculated by averaging the measure within each condition and each frequency band.

Graph analysis was used to further characterize the functional connectivity network for each condition.^11^ A minimally spanning graph, using individually set thresholds, was used to characterize the node degree of each electrode; in other words, the total number of other electrodes to which a given electrode was functionally connected. The average node degree was calculated for each channel by averaging the node degree within each condition and each frequency band.

### 2.7. Data analysis and statistics

Statistical analysis was performed using R Studio software. Differences in demographic and clinical pain characteristics in both groups were analyzed with t-testing and chi-squared tests. EEG feature variables were assessed for normality with Q-Q plots and Kolmogorov–Smirnov tests, and subsequently transformed into Z-scores. Pearson correlation analyses were performed between participant age, and PCS-C, PSQI and RCADS scores, with baseline EEG features. The EEG features of global spectral power, peak frequency, and permutation entropy across each frequency band were investigated with two-way analyses of variance of generalized linear mixed models (GLMMs) with cohort (CP and HC) and thermal stimulus (resting, tonic heat, and CPT) as fixed effects, resting-state recording type as a moderator, and participants as random effects, to account for the within-participant variability inherent to the experiment's repeated measures design. Significant main effects were analyzed using least squares means post hoc testing with Tukey corrections. Because wPLI and dPLI functional connectivity, and node degree, were calculated for each channel, they were analyzed using least squares means comparing the average channel measures during each thermal stimulus (tonic heat and CPT) with resting measures, with *P* values adjusted with the Benjamini–Hochberg procedure with a false discovery rate of 0.05. All data are presented as the mean ± standard error of the mean, unless indicated otherwise. Statistical significance was set at *P* < 0.05.

## 3. RESULTS

### 3.1. Demographic and clinical pain characteristics of participants

A total of 151 CPs and 45 HCs were recruited and completed the assessment. However, after subsequent evaluation, 2 patients did not experience pain at least once a week, and 6 patients did not have usable baseline resting-state EEG data. Therefore, 142 CPs were included in the analysis (Table 1). A higher proportion of females (83.92% vs 42.22%, *P* < 0.001) and a different distribution of ethnicity (χ^2^ = 5.67, *P* = 0.017) were observed in the CP group compared with HC group. No differences in the measures from the thermal pain modalities were identified between the groups.

**Table 1:** Demographics and Characteristics of sample.

Variable	CP (n = 143)	HC (n = 45)	Test statistic	*P*
Age, mean ± SD	14.93 ± 1.99	14.91 ± 2.23	t = 0.05	0.963
Gender, n (%)			χ^2^ = 28.75	**<0.001**
Female	120 (83.92)	19 (42.22)		
Male	23 (16.08)	26 (57.78)		
Race*, n (%)			χ^2^ = 5.67	**0.017**
White	123 (86.01)	31 (68.89)		
Person of color	20 (13.99)	14 (31.11)		
Past hospitalizations, n (%)			χ^2^ = 1.90	0.169
No	97 (67.83)	36 (80.00)		
Yes	46 (32.17)	9 (20.00)		
Past surgeries, n (%)			χ^2^ = 0.01	0.926
No	89 (62.24)	27 (60.00)		
Yes	54 (37.76)	18 (40.00)		
Dominant hand, n (%)			χ^2^ = 0.37	0.544
Left	16 (11.19)	3 (6.67)		
Right	126 (88.11)	42 (93.33)		
Primary location of pain, n (%)				
Head/neck	6 (4.20)	—		
Upper limbs	17 (11.89)	—		
Thorax	2 (1.40)	—		
Back	76 (53.15)	—		
Lower limbs	42 (29.37)	—		
Presence of radiating pain, n (%)	69 (48.25)	—		
Presence of secondary pain sites, n (%)	74 (51.75)	—		
Pain now (NRS 0–10), mean ± SD	3.60 ± 2.38	0	t = 16.22	**<0.001**
Average pain over the past month (NRS 0–10), mean ± SD	5.99 ± 1.91	—		
Worst pain over the past month (NRS 0–10), mean ± SD	8.49 ± 1.49	—		
Best pain over the past month (NRS 0–10), mean ± SD	2.03 ± 1.85	—		
Duration of pain, n (%)				
3–6 mo	19 (13.29)	—		
6–12 mo	26 (18.18)	—		
More than 12 mo	98 (68.53)	—		
Frequency of pain, n (%)				
Once a day	117 (81.82)	—		
Every second day	19 (13.29)	—		
Once a week	7 (4.90)	—		
Duration of painful episode, n (%)				
Few seconds	1 (0.70)	—		
Few minutes	17 (11.89)	—		
Few hours	30 (20.98)	—		
Constant	95 (66.43)	—		
Douleur Neuropatique 4 questionnaire				
Total score, mean ± SD	3.16 ± 1.99	—		
Likely neuropathic, n (%)	62 (44.29)	—		
Pain catastrophizing scale, mean ± SD				
Total score (x/52)	29.35 ± 10.10	17.33 ± 7.86	t = 8.31	**<0.001**
Revised Child Anxiety and Depression Scale				
Total T-score, mean ± SD	52.56 ± 13.90	44.82 ± 11.02	t = 3.84	**<0.001**
Below clinical threshold (≤64), n (%)	115 (80.42)	42 (93.33)	χ^2^ = 4.34	0.114
Borderline clinical threshold (65–69), n (%)	5 (3.50)	1 (2.22)		
Above clinical threshold (≥70), n (%)	23 (16.08)	2 (4.44)		
Pittsburgh Sleep Quality Index				
Total score (x/21), mean ± SD	7.88 ± 3.59	4.35 ± 2.48	t = 7.30	**<0.001**
Good sleep quality, n (%)	26 (18.18)	27 (60.00)	χ^2^ = 25.98	**<0.001**
Poor sleep quality, n (%)	112 (78.32)	18 (40.00)		
Tonic heat				
Pain intensity (CoVAS 0–100), mean ± SD	40.36 ± 21.29	39.22 ± 15.74	t = 0.38	0.701
Cold pressor task				
Pain intensity (NRS 0–10), mean ± SD	7.01 ± 2.45	6.26 ± 2.34	t = 1.84	0.070

Percentages do not always add up to 100% due to missing data for some demographic variables.

* Due to low frequency of some racial groups, races typically identified by Statistics Canada as a visible minority group (American Indian or Alaska Native, Asian, Black or African American, Latin American, Arab, and Mixed Race) were collapsed into a single category.

The Douleur Neuropatique 4 questionnaire was only completed by n = 140 CPs. The Pain Catastrophizing Scales was only completed by n = 142 CPs. The Pittsburgh Sleep Quality Index was only completed by n = 138 CPs.

CP, children and adolescents with chronic musculoskeletal pain; HC, age-matched healthy controls; NRS, numerical rating scale; CoVAS, computerized visual analogue scale.

### 3.2. Associations between demographic characteristics and resting electroencephalography global spectral power

**Figure 2.**
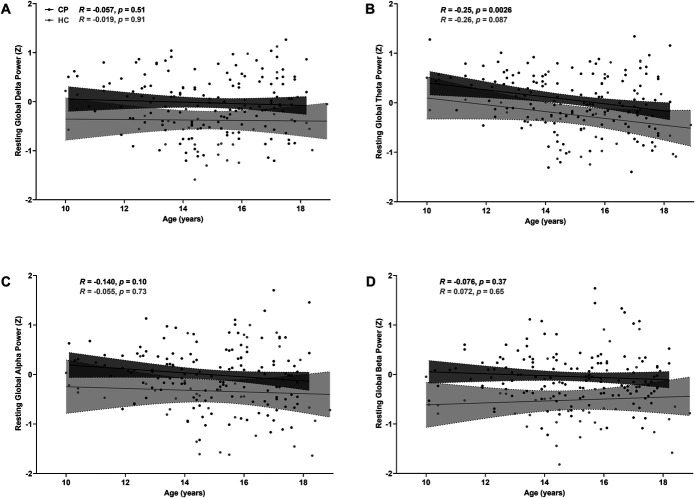
Associations between age and resting electroencephalography global delta (A), theta (B), alpha (C), and beta (D) spectral power at rest of children and adolescents with chronic musculoskeletal pain (CP) and age-matched healthy controls (HC). Pearson rank correlation analysis R values and *P* values are shown.

A significant age-related decrease in resting global theta power was only observed in the CP group (Fig. 2). No other age-related associations were observed, or between pain catastrophizing or sleep quality and resting EEG global spectral power (Figs. S1 and S2, available as supplemental digital content at http://links.lww.com/PR9/A183). However, a significant positive correlation between anxiety and depression and resting global beta power was only observed in the CP group (Fig. S3, available as supplemental digital content at http://links.lww.com/PR9/A183).

### 3.3. Changes in electroencephalography global spectral powers

**Figure 3.**
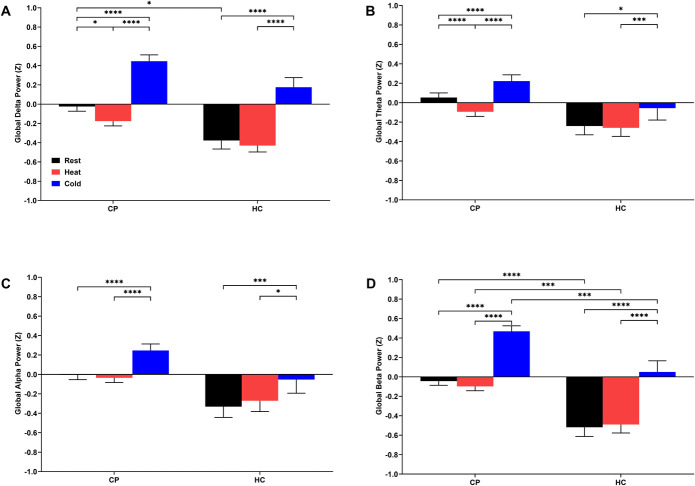
Changes in electroencephalography global delta (A), theta (B), alpha (C), and beta (D) spectral power during thermal quantitative sensory testing assessments of children and adolescents with chronic musculoskeletal pain (CP) and age-matched healthy controls (HC). Two-way ANOVAs showed main effects of cohort in the delta (F_1,179_ = 10.82, *P* = 0.0012), theta (F_1,179_ = 6.11, *P* = 0.0144), alpha (F_1,179_ = 7.19, *P* = 0.0080), and beta (F_1,179_ = 21.87, *P* < 0.0001) frequency bands, and thermal stimulus in the delta (F_2,331_ = 111.19, *P* < 0.0001), theta (F_2,331_ = 44.00, *P* < 0.0001), alpha (F_2,331_ = 40.78, *P* < 0.0001), and beta (F_2,331_ = 197.02, *P* < 0.0001) frequency bands. Statistically significant differences related to thermal quantitative sensory testing condition identified through least squares means testing with Tukey post hoc pairwise comparisons are shown by *P < 0.05, **P < 0.01, ***P < 0.005, ****P < 0.001. Data are presented as mean ± standard error of the mean (SEM).

CPs showed increased resting global delta power relative to HCs (*P* = 0.0493, Fig. 3A) but no significant cohort-related differences in global theta or alpha powers (Fig. 3B, C). Moreover, CPs showed increased global beta power relative to HCs at rest (*P* = 0.0002) and during the tonic heat (*P* = 0.0070) and cold (*P* = 0.0010) pain modalities (Fig. 3D). No significant correlation between the psychosocial characteristics and global beta power during the hot and cold modalities was observed (data not shown). A decrease in global delta (*P* = 0.0190) and theta (*P* = 0.0007) power was observed in CPs during tonic heat relative to rest (Fig. 3A, B). No differences in global spectral powers during tonic heat relative to rest were found in HCs (*P* > 0.05). In addition, CPs and HCs showed increased global spectral powers across all frequency bands during the CPT relative to rest and tonic heat (*P* < 0.05; Fig. 3A–D). In summary, CPs showed increased resting EEG global delta and beta power and a differential response of suppressed global spectral powers across delta and theta bands to tonic heat in comparison to HCs.

### 3.4. Changes in electroencephalography global peak frequencies

**Figure 4.**
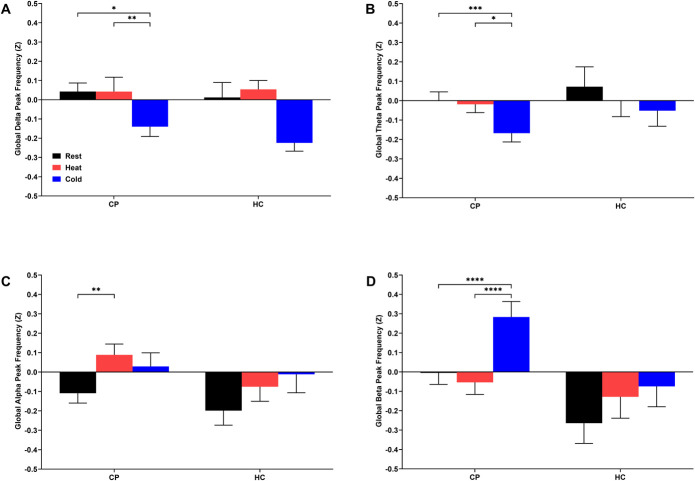
Changes in electroencephalography global peak delta (A), theta (B), alpha (C), and beta (D) frequency during thermal quantitative sensory testing assessments of children and adolescents with chronic musculoskeletal pain (CP) and age-matched healthy controls (HC). Two-way ANOVAs showed a main effect of thermal stimulus in the delta (F_2,331_ = 11.84, *P* < 0.0001), theta (F_2,331_ = 7.54, *P* = 0.0006), alpha (F_2,331_ = 7.17, *P* = 0.0009), and beta (F_2,331_ = 18.16, *P* < 0.0001) frequency bands. Statistically significant differences related to thermal quantitative sensory testing condition identified through least squares means testing with Tukey post hoc pairwise comparisons are shown by *P < 0.05, **P < 0.01, ***P < 0.005, ****P < 0.001. Data are presented as mean ± standard error of the mean (SEM).

A significant decrease in global peak delta (Fig. 4A) and theta (Fig. 4B) frequency during the CPT was observed for CPs relative to rest (*P* < 0.05) and tonic heat (*P* < 0.05). CPs showed significant increase in global peak alpha frequency during tonic heat (*P* = 0.0093; Fig. 4C) relative to rest. A significant increase in global peak beta frequency was identified in CPs during the CPT relative to rest (*P* < 0.0001) and tonic heat (*P* < 0.0001; Fig. 4D). No condition-related differences were found in HCs across all frequency bands (*P* > 0.05). In summary, only CPs showed increased global peak alpha frequency during tonic heat, decreased global peak delta and theta frequencies, and increased global peak beta frequency during the CPT.

### 3.5. Changes in electroencephalography global permutation entropy

**Figure 5.**
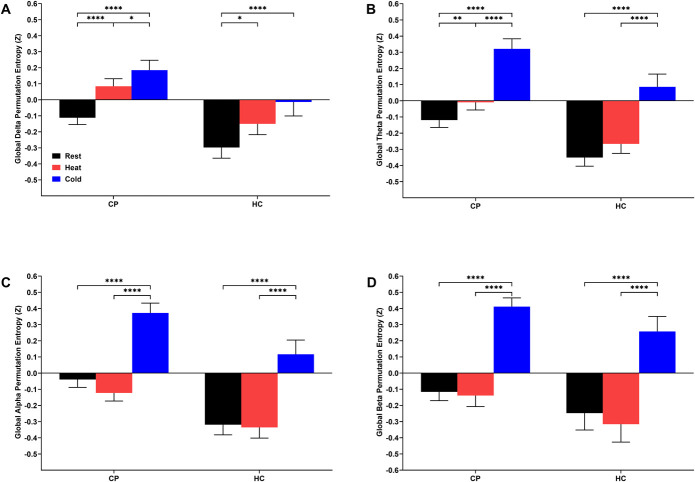
Changes in electroencephalography global delta (A), theta (B), alpha (C), and beta (D) permutation entropy during thermal quantitative sensory testing assessments of children and adolescents with chronic musculoskeletal pain (CP) and age-matched healthy controls (HC). Two-way ANOVAs showed a main effect of thermal stimulus in the delta (F_2,331_ = 48.35, *P* < 0.0001), theta (F_2,331_ = 139.66, *P* < 0.0001), alpha (F_2,331_ = 131.33,*P* < 0.0001), and beta (F_2,331_ = 112.84, *P* < 0.0001) frequency bands and a main effect of cohort in the delta (F_1,179_ = 5.22, *P* = 0.0235), theta (F_1,179_ = 7.10, *P* = 0.0084), and alpha (F_1,179_ = 7.03, *P* = 0.0088) bands. Statistically significant differences related to thermal quantitative sensory testing condition identified through least squares means testing with Tukey post hoc pairwise comparisons are shown by *P < 0.05, **P < 0.01, ***P < 0.005, ****P < 0.001. Data are presented as mean ± standard error of the mean (SEM).

Significant increases in global permutation entropy in the delta frequency band during the CPT (*P* < 0.001) and tonic heat (*P* < 0.05) relative to rest were found in CPs and HCs (Fig. 5A). However, only CPs showed increased global delta permutation entropy during the CPT relative to tonic heat (*P* = 0.045). Both CPs and HCs showed increased global permutations entropy during the CPT across the theta (*P* < 0.001; Fig. 5B), alpha (*P* < 0.001; Fig. 5C), and beta (*P* < 0.001; Fig. 5D) frequency bands relative to resting measurements and the tonic heat conditions. A significant increase in global permutation in the theta frequency band during tonic heat (*P* = 0.0051) relative to rest was only found in CPs. In summary, although the CPT elicited significant increases in global permutation entropy across the frequency bands in both groups, only CPs showed increased global delta and theta permutation entropy during tonic heat.

### 3.6. Changes in electroencephalography functional connectivity

**Figure 6.**
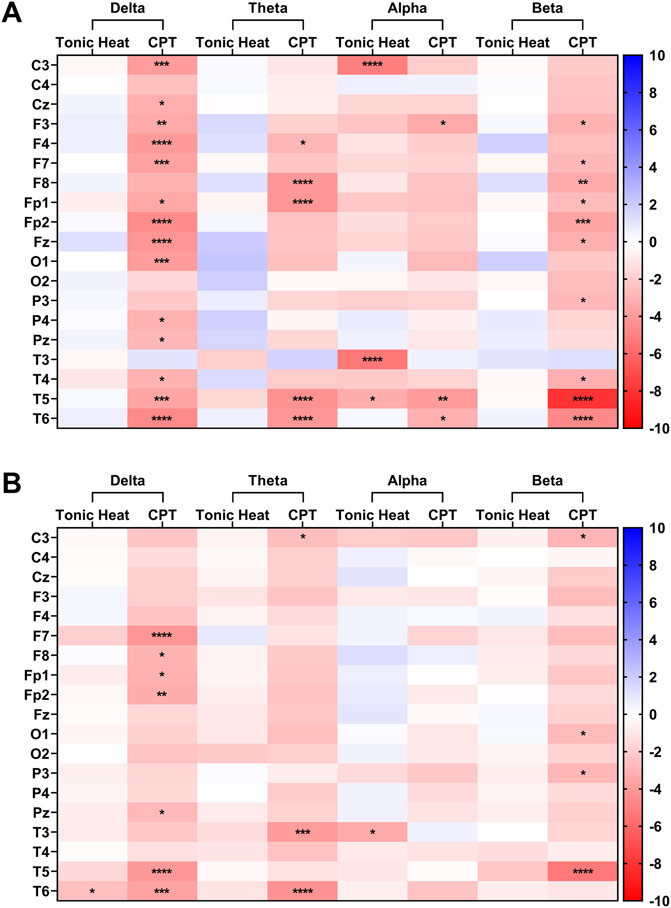
Changes in electroencephalography network functional connectivity as measured by comparing the weighted phase-lag index (wPLI) at each channel in the delta, theta, alpha, and beta frequency band during each thermal condition with resting measurements in (A) children and adolescents with chronic musculoskeletal pain and (B) age-matched healthy controls. Statistically significant differences related to thermal condition identified through least squares means testing with *P* values adjusted for multiple comparisons with the Benjamini–Hochberg procedure with an FDR of 0.05 are shown by *P < 0.05, **P < 0.01, ***P < 0.005, ****P < 0.001. Data shown are t ratios, which represent the estimate difference between the average network functional connectivity measured at rest and during the thermal condition divided by the standard error. (A) negative t ratio (displayed as red) represents an increase in network wPLI functional connectivity, whereas a positive t ratio (displayed as blue) represents a decrease in network wPLI functional connectivity. FDR, false discovery rate.

Tonic heat increased alpha wPLI connectivity of CPs at the C3, T3, and T5 channels (Fig. 6A). During the tonic heat, HCs showed increased wPLI connectivity at the T6 channel in the delta frequency band, and the T3 channel in the alpha frequency band (Fig. 6B). CPs showed significant increase in delta wPLI connectivity during the CPT globally across all channels except for C4, F8, O2, P3, and T3. The CPT significantly increased theta wPLI connectivity in the F4, F8, Fp1, T5, and T6 channels and increased alpha wPLI connectivity at the F3, T5, and T6 channels. The tonic heat condition also increased beta wPLI connectivity of CPs at the frontal F3, F7, F8, Fp1, Fp2, and Fz channels, as well as the P3, T4, T5, and T6 channels. HCs showed significant increase in wPLI connectivity during the CPT across the F7, F8, Fp1, Fp2, Fz, Pz, T5, and T6 channels in the delta frequency band, the C3, T3, and T6 channels in the theta frequency band, and the C3, O1, P3, and T5 channels in the beta frequency band.

When investigating the temporal precedence between 2 EEG signals, tonic heat increased theta dPLI connectivity of CPs at the F8 channel but decreased alpha dPLI connectivity at the T3 channel (Fig. S4A, available as supplemental digital content at http://links.lww.com/PR9/A183). During the tonic heat, HCs showed a decrease in delta wPLI connectivity at the T5 channel (Fig. S4B, available as supplemental digital content at http://links.lww.com/PR9/A183). CPs showed significant reduction in dPLI connectivity during the CPT at the P3 channel in the theta frequency band, the T5 and T6 channel in the alpha frequency band, and the T5 channel in the beta frequency band. HCs showed significant decrease in dPLI connectivity during the CPT at the O1 and T5 channels in the delta frequency band and the occipital O1, O2, T5, and T6 channels in the alpha frequency band.

In summary, CPs and HCs display significant increase in wPLI functional connectivity, but both displayed significant increase or decrease in dPLI functional connectivity across distinct EEG channels during tonic heat but especially the CPT relative to rest.

### 3.7. Changes in electroencephalography node degree

Tonic heat increased the node degree of CPs at the T3 channel in the theta frequency band and at the C3 and T3 channels in the alpha frequency band (Fig. S5A, available as supplemental digital content at http://links.lww.com/PR9/A183). HCs only showed increased node degree at the T3 channel in the alpha frequency band (Fig. S5B, available as supplemental digital content at http://links.lww.com/PR9/A183). CPs showed significant increase in node degree during the CPT at the T5 channel in the beta frequency band. In summary, CPs displayed increased node degree during the tonic heat condition in the theta and alpha frequency bands and during the CPT in the beta frequency band, whereas HCs only displayed increased node degree during the tonic heat condition in the alpha frequency band.

## 4. Discussion

### 4.1. Distinct electroencephalography spectral power in pediatric patients with chronic musculoskeletal pain

CPs showed increased resting global delta and beta power, which aligns with previous studies in adult patients with chronic neurogenic pain.^65,73^ Moreover, a significant correlation between anxiety and depression and resting global beta power was only observed in CPs. Adult studies have observed that EEG overactivation was reduced after treatment,^28,55,65,73^ and depression was associated with an increase in absolute theta and beta power.^51^ Therefore, resting global delta and beta power may have potential as a useful EEG-derived biomarker for chronic pediatric MSK pain conditions that can be targeted. In addition, although age-correlated reductions in theta and delta power have been observed in previous studies of EEG spectral power changes in healthy children,^6,26^ this trend was only found in the theta power of CPs. This lack of a well-characterized EEG developmental pattern linked to gray matter tissue loss and synaptic pruning^9,68,77^ in our HCs may be the result of a diversity of neuroplasticity processes involved in brain maturation. However, the lack of EEG developmental pattern in our CPs may provide evidence for persistent changes in central sensitivity, a key feature of chronic pain.^78^ Changes in microglial function and activity, involved in developmental synaptic pruning, elicited by the long-term release of stress hormones and immune mediators in chronic pain may underlie this EEG finding of altered brain development.^39,57^ This highlights the need for effective detection and management of persistent pain in youth to intervene against and prevent long-lasting developmental consequences.

Although no diagnosis-related differences in thermal pain responses were found, tonic heat stimulation decreased global spectral delta and theta powers and increased global peak alpha frequency in CPs but not in HCs. Global spectral power and peak alpha frequency have been shown to be negatively-^12,32,52,53,59,81^ and positively,^54^ correlated, respectively, with perceptions of tonic heat pain; thus, these observations suggest an increased thermal pain sensitivity in CPs. The CPT increased global spectral powers across all frequency bands in both CPs and HCs. However, only CPs showed decreased global peak delta and theta frequencies and increased global peak beta frequency during CPT. Taken together with evidence that peak frequency decelerations in the low-frequency bands and peak frequency accelerations in the high-frequency bands are associated with reduced pain tolerance,^5^ these observations also suggest increased sensitivity to cold pain in CPs. These observed spectral power changes between the groups extend the findings in adult patients with different chronic pain presentations.^21,61,67,69^

### 4.2. Electroencephalography waveform and functional connectivity in pediatric patients with chronic musculoskeletal pain

CPs and HCs showed increased EEG global permutation entropy across the frequency bands during the CPT; however, only CPs showed increased global delta and theta permutation entropy during tonic heat. Permutation entropy has been correlated with changes in levels of consciousness and depth of sedation because the EEG assumes a low-frequency delta wave pattern due to anesthesia.^36,56^ It may be expected that pain processes would increase permutation entropy as ascending spinal pain fibers first pass through the brain stem reticular formation, where diffuse pain-associated increased wakefulness and alertness are generated.^48^ The observation of CPs gaining high-frequency components during tonic heat provides additional evidence of increased sensitivity to thermal pain. CPs also showed a different network functional connectivity profile in response to the thermal conditions, primarily through wPLI, extending previous work in adult patients with chronic pain.^38,42^ Only CPs showed increases in network functional connectivity particularly in the beta bands of the bilateral temporal and frontal scalp channels during the CPT. Although it is difficult to draw conclusions about the underlying brain networks from EEG findings due to its low spatial resolution, this scalp distribution of beta functional connectivity roughly overlies the dorsolateral prefrontal cortex (DLPFC), primary somatosensory cortex (S1), and anterior cingulate cortex (ACC), which is consistent with previous adult studies observing altered resting functional connectivity in the PFC and the ACC.^49,75^ The brain regions with observed increase in functional connectivity are implicated in sensory pain processing pathways and the circuits mediating the affective and cognitive aspects of chronic pain.^13,69,79^. Because prefrontal regions mediate executive control functions which permit cognitive reappraisals of pain and pain-associated emotions^33,70^ and that stimulating the ACC may attenuate the emotional component of pain unpleasantness,^8^ dysregulations in the signalling within their inhibitory circuits may increase engagement of subcortical limbic regions such as the amygdala manifesting maladaptive responses to pain stimuli.^34^ With the increased S1 functional connectivity, these alterations in network functional connectivity support the hypothesis that chronic pediatric MSK pain is mediated and maintained by a dysfunctional reorganization in brain signalling patterns that shifts from the superficial brain regions primarily encoding pain sensation to subcortical regions encoding pain emotionality.^3,29,79^ In addition, diffuse suppressions of alpha network functional connectivity were found during tonic heat stimulations only in CPs. These changes in permutation entropy and functional connectivity EEG measures suggest that dynamic perturbations in the flow of information in the brain connectome underlie the sensory, affective, and cognitive pain experiences of youth with chronic MSK pain undergoing the thermal pain modalities.

### 4.3. Clinical implications

The observed EEG changes in response to tonic heat and cold stimuli, despite no differences in the thermal pain assessment, suggest that EEG is a low-cost, clinical-accessible, and noninvasive brain imaging tool.^62^ Moreover, the differential brain activity changes observed between CPs and HCs suggest that EEG may be more sensitive to the detection and interpretation of the pain mechanisms underlying pediatric chronic MSK pain than the thermal pain modalities.^20^ EEG may enhance the clinical pain assessment of children and adolescents with suspected or diagnosed chronic MSK pain conditions, particularly those with intellectual and/or developmental disabilities. Future prospective cohort studies identifying whether pharmacological or behavioural pain management influences or normalizes the perturbed brain signalling patterns observed in this study is warranted. In addition, this study identified that tonic heat and cold pain stimuli produced divergent EEG power spectra, waveform, and functional connectivity changes, suggesting that EEG may be sensitive in interrogating differences in the pain experience that are elicited by distinct experimental noxious modalities.

### 4.4. Limitations and conclusions

There were several limitations. First, cohort composition differences may have introduced confounders because the pain experience is modified by a spectrum of biopsychosocial factors. However, exploratory GLMMs showed that sex and ethnicity were statistically insignificant fixed factors (*P* > 0.05; data not shown), and previous studies have observed fair to moderate test–retest reliability in EEG recordings in different samples of youth.^43,45,64^ Second, the heterogenous composition of our CPs, with representation from a diversity of pain diagnoses, locations, severities, and neuropathic-like characteristics, may have reduced our likelihood to detect differences between the CPs and the HCs. However, our sample's heterogeneity promotes the external validity of our findings to clinicians caring for diverse chronic MSK pain clinical presentations. Third, our EEG findings could only infer the specific neurological substrates that may be responsible for the observed EEG cortical activity patterns. Future application of low-resolution brain electromagnetic tomography (LORETA) could estimate the source localization of brain electrical activity underlying the observed EEG scalp recordings during the thermal conditions.^58^ Fourth, the different baseline conditions may have influenced the significance of our results. However, this difference was statistically controlled. A notable strength of this study is its large sample size. Although a priori sample size calculation was not performed because of the paucity of effect sizes and variances reported in the EEG literature,^40^ this study's statistical power qualitatively exceeds that of most previously published EEG studies with sample sizes typically between 10 and 20 participants per cohort.

In this study, children and adolescents with chronic MSK pain and age-matched pain-free controls showed differences in resting EEG features and differential changes in EEG activity while undergoing thermal experimental pain modalities. Continuous EEG enhances the ability of thermal modalities to reveal the underlying pain mechanisms and detect changes in pain sensitivity in youth with chronic MSK pain.

## Disclosures

The authors have no conflicts of interest to declare.

## Appendix A. Supplemental digital content

Supplemental digital content associated with this article can be found online at http://links.lww.com/PR9/A183.

## Supplementary Material

SUPPLEMENTARY MATERIAL
