# Supplementary material for: Electroencephalographic characteristics of children and adolescents with chronic musculoskeletal pain
Source: Pain Rep. 2022 Dec 22;7(6):e1054. doi: 10.1097/PR9.0000000000001054 (PMC9788982; doi:10.1097/PR9.0000000000001054)
Supplement: SUPPLEMENTARY MATERIAL [file painreports-7-e1054-s001.pdf]

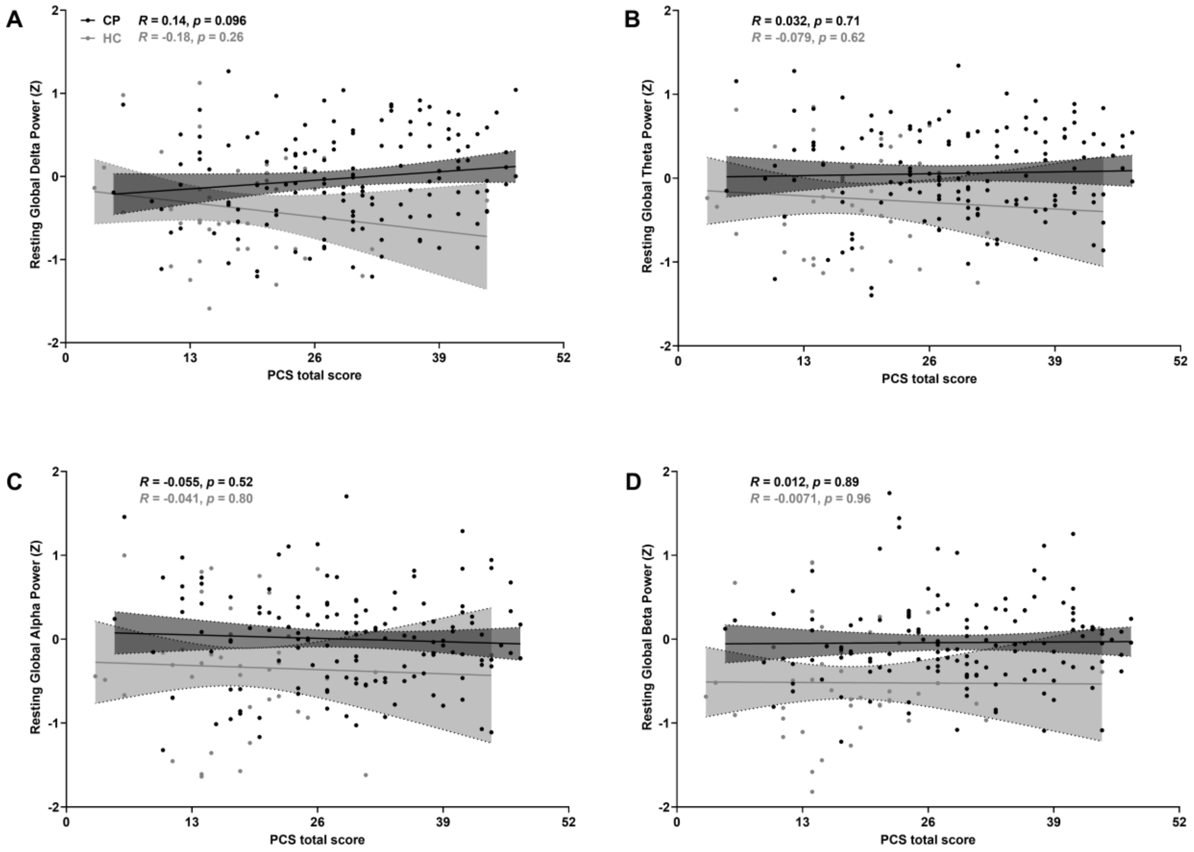

Figure S1: Associations between the total score of the pain catastrophizing scale (PCS) and resting EEG global delta (A), theta (B), alpha (C), and beta (D) spectral power at rest of children and adolescents with chronic MSK pain (CP) and age-matched healthy controls (HC). Pearson's rank correlation analysis R values and p-values are shown.

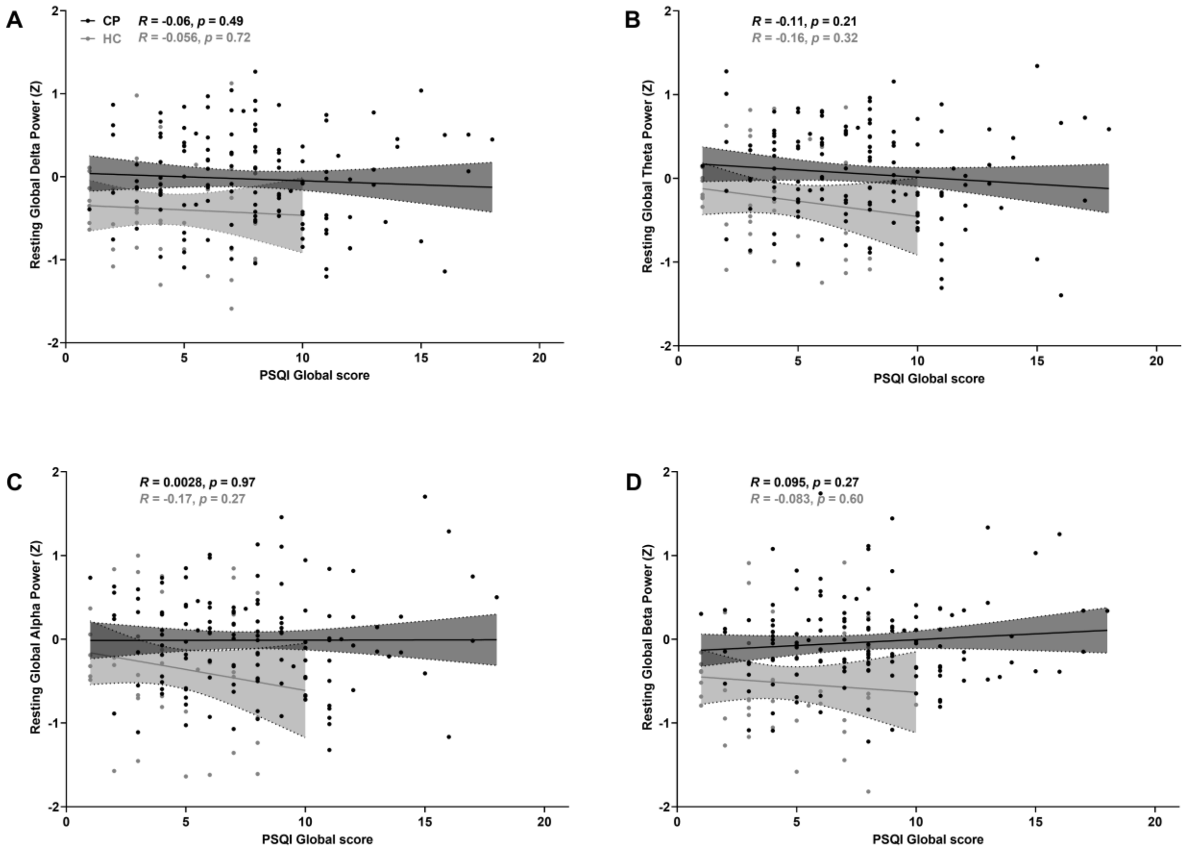

Figure S2: Associations between the global score of the Pittsburgh sleep quality index (PSQI) and resting EEG global delta (A), theta (B), alpha (C), and beta (D) spectral power at rest of children and adolescents with chronic MSK pain (CP) and age-matched healthy controls (HC). Pearson's rank correlation analysis R values and p-values are shown.

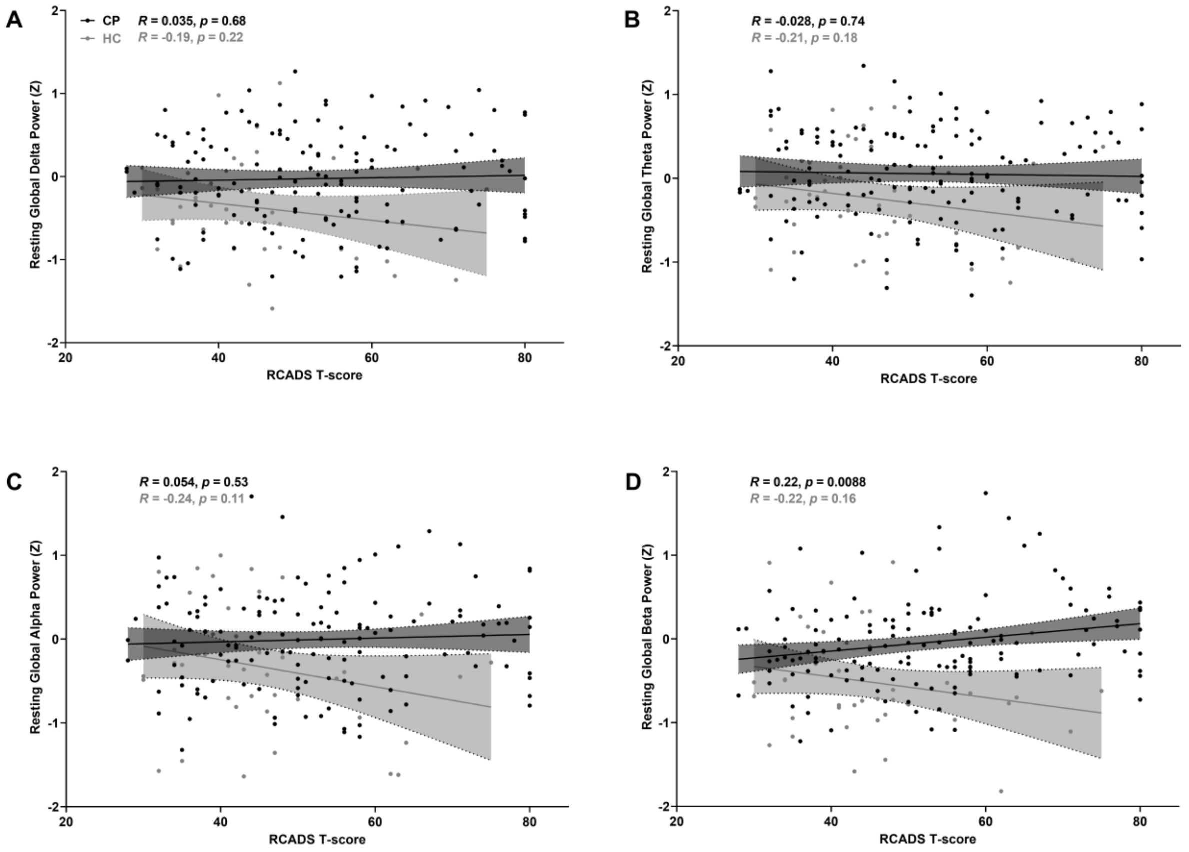

Figure S3: Associations between the T-score of the revised child anxiety and depression scale (RCADS) and resting EEG global delta (A), theta (B), alpha (C), and beta (D) spectral power at rest of children and adolescents with chronic MSK pain (CP) and age-matched healthy controls (HC). Pearson's rank correlation analysis R values and p-values are shown.

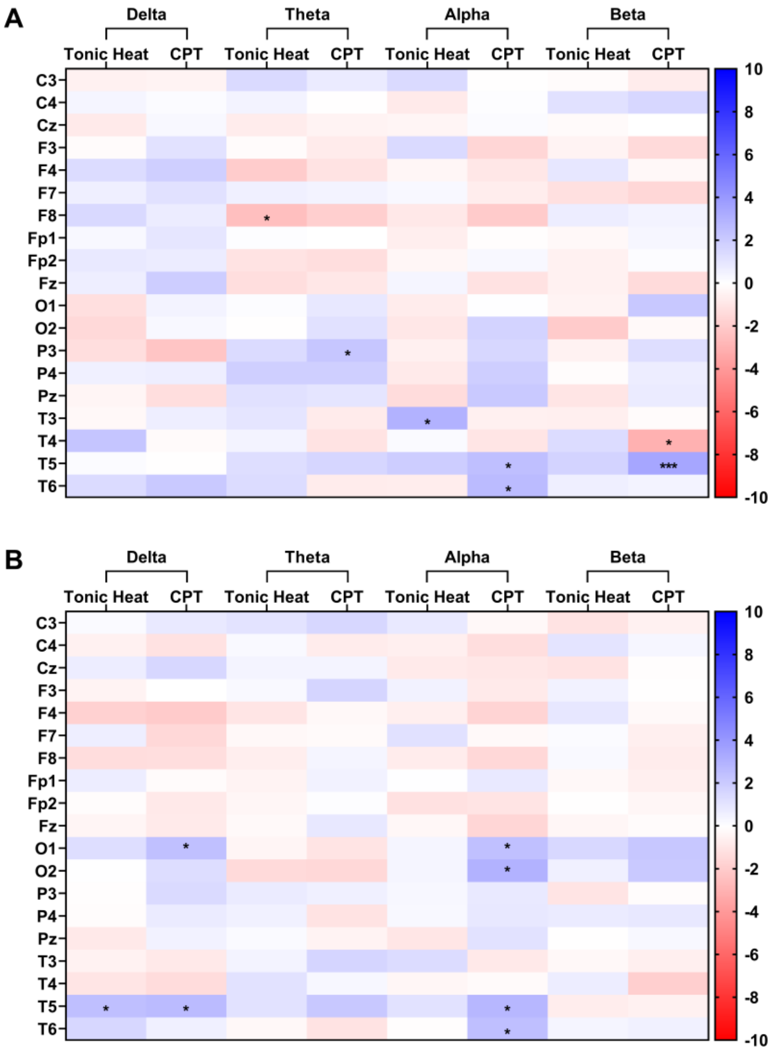

Figure S4. Changes in EEG network functional connectivity as measured by comparing the directed phase-lag index (dPLI) at each channel in the delta, theta, alpha, and beta frequency band during each thermal condition with resting measurements in (A) children and adolescents with chronic MSK pain and (B) age-matched healthy controls. Statistically significant differences related to thermal condition identified through least squares means testing with p-values adjusted for multiple comparisons with the Benjamini-Hochberg procedure with a FDR of 0.05 are shown by *p<0.05, **p<0.01, ***p<0.005, ****p<0.001. Data shown are t ratios, which represent the estimate difference between the average network functional connectivity measured at rest and during the thermal condition divided by the standard error. A negative t ratio (displayed as red) represents an increase in network dPLI functional connectivity, while a positive t ratio (displayed as blue) represents a decrease in network dPLI functional connectivity.

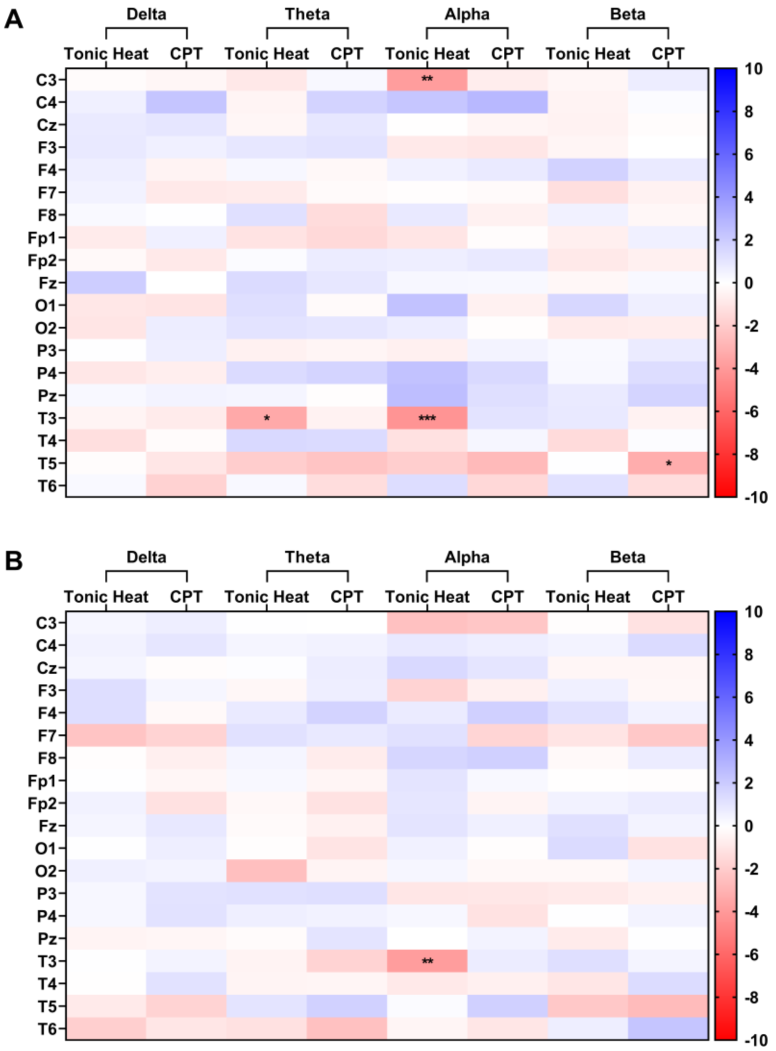

Figure S5. Changes in EEG network functional connectivity as measured by comparing the node degree at each channel in the delta, theta, alpha, and beta frequency band during each thermal condition with resting measurements in (A) children and adolescents with chronic MSK pain and (B) age-matched healthy controls. Statistically significant differences related to thermal condition identified through least squares means testing with p-values adjusted for multiple comparisons with the Benjamini-Hochberg procedure with a FDR of 0.05 are shown by *p<0.05, **p<0.01, ***p<0.005, ****p<0.001. Data shown are t ratios, which represent the estimate difference between the average network functional connectivity measured at rest and during the thermal condition divided by the standard error. A negative t ratio (displayed as red) represents an increase in network node degree, while a positive t ratio (displayed as blue) represents a decrease in network node degree.
